# Improving Biometric-Based Authentication Schemes with Smart Card Revocation/Reissue for Wireless Sensor Networks

**DOI:** 10.3390/s17050940

**Published:** 2017-04-25

**Authors:** Jongho Moon, Donghoon Lee, Youngsook Lee, Dongho Won

**Affiliations:** 1 Department of Electrical and Computer Engineering, Sungkyunkwan University, 2066 Seobu-ro, Jangan-gu, Suwon-si, Gyeonggi-do 16419, Korea; jhmoon@security.re.kr (J.M.); dhlee@security.re.kr (D.L.); 2 Department of Cyber Security, Howon University, 64 Howondae 3-gil, Impi-myeon, Gunsan-si, Jeonrabuk-do 54058, Korea; ysooklee@howon.ac.kr; 3 Department of Computer Engineering, Sungkyunkwan University, 2066 Seobu-ro, Jangan-gu, Suwon-si, Gyeonggi-do 16419, Korea

**Keywords:** wireless sensor networks, user authentication, biometric, smart card

## Abstract

User authentication in wireless sensor networks is more difficult than in traditional networks owing to sensor network characteristics such as unreliable communication, limited resources, and unattended operation. For these reasons, various authentication schemes have been proposed to provide secure and efficient communication. In 2016, Park et al. proposed a secure biometric-based authentication scheme with smart card revocation/reissue for wireless sensor networks. However, we found that their scheme was still insecure against impersonation attack, and had a problem in the smart card revocation/reissue phase. In this paper, we show how an adversary can impersonate a legitimate user or sensor node, illegal smart card revocation/reissue and prove that Park et al.’s scheme fails to provide revocation/reissue. In addition, we propose an enhanced scheme that provides efficiency, as well as anonymity and security. Finally, we provide security and performance analysis between previous schemes and the proposed scheme, and provide formal analysis based on the random oracle model. The results prove that the proposed scheme can solve the weaknesses of impersonation attack and other security flaws in the security analysis section. Furthermore, performance analysis shows that the computational cost is lower than the previous scheme.

## 1. Introduction

Wireless Sensor Networks (WSNs) generally consist of gateways, users, and a large number of sensor nodes. The sensor node is tiny and can be easily deployed in various kinds of severe environments. However, each sensor node has limited resources, is lacking in memory, has low computational capabilities and short radio transmission range [1]. Data collected from sensor nodes in WSNs sometimes include valuable and classified information such as the environmental surrounding the wartime, a patient’s personal information, monitoring information of museums, and the power company’s voltage variation monitoring data. To ensure the confidentiality and reliability of deployed WSNs, only registered and legitimate users should be able to access them. In addition, establishing secure protocol actively requires a mutual authentication between the user and the sensor node. For these reasons, secure user authentication is one of the major issues in WSNs.

To support message confidentiality and secure authentication for sensor networks, various authentication schemes for WSNs have been proposed. However, a problem that occurs with respect to password-based authentication schemes is that a server must maintain a password table to legitimately verify a login user. Therefore, the server requires additional memory space to store the password table. For this reason, many researchers have proposed a new type of remote user authentication scheme whereby personal biological characteristics are used, such as a fingerprint or an iris. The main advantage biometrics is uniqueness. A smart card can be used as a tool to store biometric information. Since the smart card has its own calculation function, it can operate at more than one level. In 2004, Watro et al. [2] proposed a user authentication scheme using the RSA and DH algorithms for WSNs. After that, Wong et al. [3] proposed a hash-based dynamic user authentication scheme in 2006. However, Tseng et al. [4] found that Wong et al.’s authentication scheme was vulnerable to replay, stolen verifiers, and forgery attacks. In 2009, Das [5] proposed and claimed that his scheme can resist various real-time attacks. Unfortunately, He et al. [6] found that Das’s scheme was vulnerable to insider and impersonation attacks, and proposed an improved two factor scheme to solve these security problems. In the same year, Khan and Alghathbar [7] demonstrated that Das’s scheme did not provide mutual authentication, and was vulnerable to gateway bypassing and privileged insider attacks, and Chen et al. [8] also found that Das’s scheme did not provide mutual authentication between the gateway and the sensor, proposed a robust mutual authentication scheme for WSNs, and claimed that their scheme provides greater security than Das’s scheme. In 2010, Yuan et al. [9] proposed a biometric-based user authentication scheme. However, Yoon et al. demonstrated that Yuan et al.’s scheme was vulnerable to insiders, user impersonation, gateway node impersonation and sensor node impersonation attacks. To solve these problems, Yoon et al. [10] proposed an improved user authentication scheme. Unfortunately, He et al. [11] found that Yoon et al.’s scheme was still vulnerable to denial of service (DoS) and sensor impersonation attacks, and then proposed an improved scheme to overcome these security problems.

In 2013, Yoon and Kim [12] pointed out that He et al.’s scheme had various security vulnerabilities such as poor repairability, and was vulnerable to user and sensor node impersonation attacks. They then proposed an advanced biometrics-based user authentication scheme for WSNs. They claimed that their scheme was more effective and had stronger security than other related schemes. However, Choi et al. [13] found that Yoon and Kim’s scheme [12] had various security problems, including a biometric recognition error, a user verification problem, lack of anonymity, perfect forward secrecy, session key exposure by the gateway node, vulnerability to DoS attacks, and a revocation problem. To overcome these vulnerabilities, they proposed a biometric-based user authentication scheme using a fuzzy extractor, and claimed that their scheme is more secure than other authentication schemes. Unfortunately, Park et al. [14] demonstrated that Choi et al.’s scheme [13] was still insecure against user impersonation attacks, and had security weakness in the revocation/reissue phase. They then proposed an enhanced biometric-based authentication scheme for WSNs that has improved security functions. After careful analysis, however, we found that Park et al.’s scheme [14] was still insecure against impersonation attack, and also had a problem in the smart card revocation/reissue phase.

In this paper, we show how an adversary can impersonate a legitimate user or a sensor node, and perform an illegal smart card revocation/reissue. After demonstrating these problems, we propose an improved biometric authentication scheme. Finally, we analyze our proposed scheme for security properties and computational cost. The proposed scheme is more secure and efficient than other related authentication schemes.

The rest of this paper is organized as follows. In Section 2, we briefly introduce the elliptic curve cryptosystem, threat assumptions and fuzzy extractors that we adopt in our scheme. In Section 3 and Section 4, we review and analyze, respectively, Park et al.’s scheme [14]. In Section 5, we propose an improved authentication scheme for WSNs. In Section 6, we present a security analysis of the proposed scheme. Section 7 shows performance analysis comparing our scheme with previous schemes. Our conclusions are presented in Section 8.

## 2. Preliminaries

In this section, we briefly introduce the elliptic curve cryptosystem, threat assumptions, and fuzzy extractor.

### 2.1. Elliptic Curve Cryptosystem

The elliptic curve cryptosystem (ECC) was first proposed by Koblitz [15] and Miller [16] to design public key cryptosystems, and presently it is widely used in several cryptographic schemes to provide desired levels of security and performance. An elliptic curve $E_{K}$ defined over a field *K* of the characteristic ≠ 2 or 3 is the set of solutions (x, y)∈$K^{2}$ to the equation:

(1) $$y^{2}=x^{3}+ax+b,\;a,b\in K,4a^{3}+27b^{2}\neq 0.$$

Cryptosystems based on GF(${q)}^{*}$ can be translated to systems using the group *E*, where *E* is an elliptic curve defined over a GF(*q*). The point multiplications kP = (P+P+...+P, *k* times) that means *k* times addition of point *P*. Given an elliptic curve *E* defined over a GF(*q*) and two points *P*, *Q* ∈ *E*, it finds an integer *x* such that Q=xP, if such *x* exists. This problem proved to be more intractable than the typically discrete logarithm problem. The details of the ECC definitions can be found in [15]. There are several computational problems based on ECC which are presented below:

**Definition** **1.** The elliptic curve discrete logarithm problem (ECDLP) is defined as: given two elements *Q*, $R\in G_{p}$, find an integer k∈[1, n−1] such that R=kQ.

**Definition** **2.** The computational Diffie–Hellman problem (CDHP) is defined as: given three elements (P, aP, bP) for any *a*, b∈[1, n−1], computation of abP is very hard to the group $G_{p}$.

**Definition** **3.** The elliptic curve factorization problem (EC FP) is defined as: given two elements *P*, $Q\in G_{p}$, where Q=sP+tP and (s, t)∈[1, n−1], then computation of sP and tP is impossible.

**Definition** **4.** The decisional Diffie–Hellman problem (DD HP) is defined as: given four elements (P, aP, bP, cP) for any (a, *b*, c) ∈[1, n−1], decide whether or not cP=abP i.e., c=ab mod*p* or not.

**Definition** **5.** The weak Diffie–Hellman problem (WDHP) is defined as: for $Q\in G_{p}$ and some k∈[1, n−1] from the given three elements (P, *Q*, kP), computation of kQ is very hard.

### 2.2. Threat Assumptions

We introduce the Dolev–Yao [17] and some threat model [18,19], and consider the risk of side channel attack [20] to construct the threat assumptions, which are described as follows:

1. An adversary A can be either a user, gateway, or sensor node. Any registered user can act as an adversary.
2. An adversary A can eavesdrop every communication in a public channel, thereby capturing any message exchanged between a user and gateway or sensor node.
3. An adversary A has the ability to alter, delete or reroute the captured message.
4. Information can be extracted from the smart card by examining the power consumption of the card.

### 2.3. Fuzzy Extractor

In this subsection, we describe the basis for a biometric-based fuzzy extractor, which converts biometric information data into a random value. Based on [21,22,23], the fuzzy extractor is given by two procedures (Gen, Rep). The mechanism of a fuzzy extractor consists of two procedures (Gen, Rep), which is demonstrated as:

- $Gen\left(BIO\right)\to \langle R,P\rangle ,$
- $Rep(BIO^{*},P)=R$ if $BIO^{*}$ is reasonably close to BIO.

The function Gen is a probabilistic generation procedure, which on biometric input BIO outputs an “extracted” string $R\in {\left\{0,1\right\}}^{l}$ and an auxiliary string $P\in {\left\{0,1\right\}}^{*}$. Rep is a deterministic reproduction procedure, which recovery of *R* from the corresponding auxiliary string *P*, and any vector $BIO^{*}$ close to BIO. The detailed information about fuzzy extractors can be founded in the literature [24].

## 3. Review of Park et al.’s Authentication Scheme

In this section, we review Park et al.’s authentication scheme [14]. The notations used in the paper are listed in Table 1. The GW generates two master keys, *x* and *y*, and provides a long-term secret key $h(SID_{j}||y)$ to the sensor $S_{j}$ before the scheme is executed. Their scheme involves three parties, i.e., the user $U_{i}$, the gateway GW and the sensor $S_{j}$, to communicate with each other to perform the following three phases: registration, login/authentication, and revocation/reissue.

### 3.1. Registration Phase

In this phase, a user $U_{i}$ chooses an identity $ID_{i}$, imprints biometric template $B_{i}$, and then performs the following steps:

1. $U_{i}$ computes $\langle R_{i}$, $P_{i}\rangle =Gen\left(B_{i}\right)$ and $A_{i}=h\left(R_{i}\right)$. $U_{i}$ then sends $\langle ID_{i},A_{i}\rangle$ to the gateway GW.
2. Upon receiving $\langle ID_{i}$, $A_{i}\rangle$ from $U_{i}$, GW computes the authentication parameters as:
  $$V_{i}=h(ID_{i}\left|\right|A_{i})\;M_{i}=h(x||y||A_{i})\;N_{i}=M_{i}⊕A_{i}\;C_{i}=E_{x}(A_{i}||up_{i}).$$
3. GW stores h(·) and the authentication parameters; $\langle V_{i}$, $N_{i}$, $C_{i}$, h(·)〉 in the smart card $SC_{i}$. GW then issues $SC_{i}$ to $U_{i}$ through a secure channel.
4. After receiving the smart card from GW, $U_{i}$ stores $P_{i}$ in the smart card.

### 3.2. Login and Authentication Phase

If a user $U_{i}$ wants to log in to the GW and $S_{j}$, $U_{i}$ performs the login phase, and then $U_{i}$, GW and $S_{j}$ verify each other’s authenticities. Finally, $U_{i}$ and $S_{j}$ generate a session key in this phase as follows:

1. $U_{i}$ enters $ID_{i}$ and imprints biometric template $B_{i}^{*}$. $SC_{i}$ then computes $R_{i}^{*}$, $A_{i}^{*}$ and $V_{i}^{*}$ using fuzzy extraction, and compares $V_{i}^{*}$ with $V_{i}$ as:
  $$R_{i}^{*}=Rep\left(B_{i}^{*},P_{i}\right)\;A_{i}^{*}=h\left(R_{i}^{*}\right)\;V_{i}^{*}=h(ID_{i}\left|\right|A_{i}^{*})\;verifies\;V_{i}\overset{?}{=}V_{i}^{*}.$$
2. $SC_{i}$ generates a random number $r_{i}$ and computes $X_{i}$ and $M_{i}$ as:
  $$X_{i}=r_{i}\times P\;M_{i}=N_{i}⊕A_{i}.$$
3. $U_{i}$ picks up $T_{i}$ and computes $AID_{i}$ and $W_{i}$ as:
  $$AID_{i}=ID_{i}⊕h(M_{i}\left|\right|T_{i})\;W_{i}=h(M_{i}||ID_{i}\left|\right|X_{i}\left|\right|T_{i}).$$
  $U_{i}$ then sends the login request message $M_{1}=\langle AID_{i}$, $X_{i}$, $C_{i}$, $T_{i}$, $W_{i}\rangle$ to GW.
4. After receiving $M_{1}$, GW retrieves $T^{\prime }$ and verifies $T^{\prime }-T_{i}\leq △T$. If this holds, GW computes $A_{i}^{*}$, $M_{i}^{*}$, $ID_{i}^{*}$ and $W_{i}^{*}$, and compares $W_{i}^{*}$ with $W_{i}$ as:
  $$A_{i}^{*}\left|\right|up_{i}=D_{x}\left(C_{i}\right)\;M_{i}^{*}=h(x||y||A_{i}^{*})\;ID_{i}^{*}=AID_{i}⊕h(M_{i}^{*}\left|\right|T_{i})\;W_{i}^{*}=h(M_{i}^{*}||ID_{i}^{*}\left|\right|X_{i}^{*}\left|\right|T_{i})\;verifies\;W_{i}\overset{?}{=}W_{i}^{*}.$$
  If this holds, GW verifies the legitimacy of $U_{i}$.
5. GW picks up $T_{g}$ and computes $k_{g}$, $C_{g}$ and $W_{g}$,
  $$k_{g}=h(h(SID_{j}\left||y)|\right|T_{g})\;C_{g}=E_{k_{g}}(AID_{i}\left|\right|X_{i})\;W_{g}=h(h(SID_{j}||y)||AID_{i}\left|\right|C_{g}\left|\right|T_{g}).$$
  GW then sends the authentication message $M_{2}=\langle AID_{i}$, $C_{g}$, $T_{g}$, $W_{g}\rangle$ to $S_{j}$.
6. When receiving $M_{2}$ from GW, $S_{j}$ retrieves $T^{\prime \prime }$, and verifies $T^{\prime \prime }-T_{g}\leq △T$. If this holds, $S_{j}$ verifies the validity of $W_{g}$ by comparing it with $h(h(SID_{j}||y)||AID_{i}\left|\right|C_{g}\left|\right|T_{g})$ to check the legitimacy of GW. After that, $S_{j}$ computes $k_{g}^{*}$, and decrypts $C_{g}$ using $k_{g}^{*}$. $S_{j}$ then checks the validity of the received $AID_{i}$ by comparing $AID_{i}^{*}$ as:
  $$k_{g}^{*}=h(h(SID_{j}\left||y)|\right|T_{g})\;D_{k_{g}^{*}}=AID_{i}^{*}\left|\right|X_{i}^{*}\;verifies\;AID_{i}\overset{?}{=}AID_{i}^{*}.$$
7. $S_{j}$ generates a random number $r_{s}$ and computes $K_{SU}$, $Y_{i}$ and a session key sk. $S_{j}$ then picks up $T_{s}$ and computes RM, $V_{s}$ as:
  $$K_{SU}=r_{x}\times X_{i}^{*}\;Y_{i}=r_{s}\times P\;sk=h(AID_{i}||K_{SU}||T_{s})\;RM=\text{Response to the query of }U_{i}\;V_{s}=h(AID_{i}\left|\right|X_{i}\left|\right|Y_{i}\left||RM|\right|T_{s}).$$
  $S_{j}$ hence sends $M_{3}=\langle RM$, $Y_{i}$, $V_{s}$, $T_{s}\rangle$ to $U_{i}$.
8. Upon receiving $M_{3}$, $U_{i}$ retrieves $T_{s}$, and checks the sameness of $V_{s}$. $U_{i}$ then computes $K_{US}$ and sk as:
  $$V_{s}^{*}=h(AID_{i}\left|\right|X_{i}\left|\right|Y_{i}\left||RM|\right|T_{s})\;verifies\;V_{s}^{*}\overset{?}{=}V_{s}\;K_{US}=r_{i}\times Y_{i}\;sk=h(AID_{i}||K_{US}||T_{s}).$$
  Only the legitimate $U_{i}$ can compute $K_{US}$ and sk. $U_{i}$ then accepts RM. Finally, $U_{i}$ and $S_{j}$ can communicate securely using the common sk.

### 3.3. Revocation or Reissue Phase

To make up for smart card loss or long term key disclosure, the smart card should be revoked and reissued in cycles.

1. $U_{i}$ who wants to revoke and reissue a smart card inputs the previous identity $ID_{i}$ and the new identity $ID_{i}^{*}$ to prevent adversaries from registering with the same identity $ID_{i}$. Then, $U_{i}$ imprints biometric template $B_{i}$ and computes $R_{i}=Rep\left(B_{i},P_{i}\right)$, $A_{i}=h\left(R_{i}\right)$ and $M_{i}=N_{i}⊕A_{i}$.
2. $U_{i}$ computes $Z_{i}=ID_{i}⊕M_{i}$ and sends the revocation/reissue request message $\langle ID_{i}$, $ID_{i}^{*}$, $A_{i}$, $Z_{i}\rangle$ to GW.
3. GW computes $M_{i}^{*}$, $Z_{i}^{*}$ and checks the legitimacy of the user as:
  $$M_{i}^{*}=h(x||y||A_{i})\;Z_{i}^{*}=ID_{i}⊕M_{i}^{*}\;verifies\;Z_{i}^{*}\overset{?}{=}Z_{i}.$$
4. If this holds, GW revokes $ID_{i}$ and records it on the revocation look-up table. Then, GW computes new authentication parameters $V_{i}$, $N_{i}$ and $C_{i}$ as:
  $$V_{i}=h(ID_{i}^{*}\left|\right|A_{i})\;N_{i}=M_{i}^{*}⊕A_{i}\;C_{i}=E_{x}(A_{i}||up_{i}^{*}).$$
5. GW stores h(·) and the new authentication parameters; $\langle V_{i}$, $N_{i}$, $C_{i}$, h(·)〉 in the smart card $SC_{i}$. GW then reissues $SC_{i}$ to $U_{i}$ through a secure channel.
6. $U_{i}$ stores $P_{i}$ in the smart card.

## 4. Cryptanalysis of Park et al.’s Authentication Scheme

In this section, we analyze the security problems of Park et al.’s scheme [14]. Park et al. cryptanalyzed a scheme of Choi et al. [13] and improved it to support better security functionality. However, we found that Park et al.’s scheme [14] was still insecure against impersonation attack and had a problem with smart card revocation/reissue. The following attacks are based on the threat assumptions that a malicious adversary A was completely monitored through the communication channel connecting $U_{i}$, GW and $S_{j}$ in the login and authentication phases, and that the A obtained the information saved in their own smart card [20]. A therefore can eavesdrop, modify, insert, or delete any message transmitted over a public channel. We now reveal the details of these problems.

### 4.1. User Impersonation Attack

1. Let A be an active adversary who is he/she legal user and owns a smart card to extract information $\langle V_{a}$, $N_{a}$, $C_{a}$, h(·), $P_{a}\rangle$.
2. A then imprints one’s biometric template $B_{a}^{*}$ and computes $R_{a}^{*}=Rep\left(B_{a}^{*},P_{a}\right)$ and $A_{a}^{*}=h\left(R_{a}^{*}\right)$.
3. A generates a random number $r_{A}$, and selects any identity $ID_{i}$. A then computes login request message $M_{1}$ as:
  $$X_{a}=r_{a}\times P\;M_{a}=N_{a}⊕A_{a}^{*}\;AID_{i}=ID_{i}⊕h(M_{a}\left|\right|T_{a})\;W_{a}=h(M_{a}||ID_{i}\left|\right|X_{a}\left|\right|T_{a}).$$
  A then sends the login request message $M_{1}=$ $\langle AID_{i}$, $X_{a}$, $C_{a}$, $T_{a}$, $W_{a}\rangle$ to GW.
4. When receiving $M_{1}$, GW retrieves $T^{\prime }$ and verifies $T^{\prime }-T_{a}\leq △T$. If this holds, GW computes $A_{a}^{*}$, $M_{a}^{*}$, $ID_{i}^{*}$, $W_{a}^{*}$ and compares $W_{a}^{*}$ with $W_{a}$ as:
  $$A_{a}^{*}\left|\right|up_{a}=D_{x}\left(C_{a}\right)\;M_{a}^{*}=h(x||y||A_{a}^{*})\;ID_{i}^{*}=AID_{i}⊕h(M_{a}^{*}\left|\right|T_{a})\;W_{a}^{*}=h(M_{a}^{*}||ID_{i}^{*}\left|\right|X_{a}^{*}\left|\right|T_{a})\;verifies\;W_{a}\overset{?}{=}W_{a}^{*}.$$
  If this holds and $ID_{i}$ does exist in the database, the gateway GW continues to proceed the scheme without detected. Otherwise, A selects another identity nominee and performs the same processes until he/she locates the valid identity.
5. GW picks up $T_{g}$ and computes $k_{g}$, $C_{g}$ and $W_{g}$:
  $$k_{g}=h(h(SID_{j}\left||y)|\right|T_{g})\;C_{g}=E_{k_{g}}(AID_{i}\left|\right|X_{a})\;W_{g}=h(h(SID_{j}||y)||AID_{i}\left|\right|C_{g}\left|\right|T_{g}).$$
  GW then sends the authentication message $M_{2}=$ $\langle AID_{i}$, $C_{g}$, $T_{g}$, $W_{g}\rangle$ to $S_{j}$.
6. Upon receiving $M_{2}$ from GW, $S_{j}$ retrieves $T^{\prime \prime }$ and verifies $T^{\prime \prime }-T_{g}\leq △T$. If this holds, $S_{j}$ verifies the validity of $W_{g}$ by comparing it with $h(h(SID_{j}||y)||AID_{i}\left|\right|C_{g}\left|\right|T_{g})$ to check the legitimacy of GW. After that, $S_{j}$ computes $k_{g}^{*}$ and decrypts $C_{g}$ using $k_{g}^{*}$. $S_{j}$ then checks the validity of the received $AID_{i}$ by comparing $AID_{i}^{*}$ as
  $$k_{g}^{*}=h(h(SID_{j}\left||y)|\right|T_{g})\;D_{k_{g}^{*}}=AID_{i}^{*}\left|\right|X_{a}^{*}\;verifies\;AID_{i}\overset{?}{=}AID_{i}^{*}.$$
7. $S_{j}$ generates a random number $r_{s}$ and computes $K_{SU}$, $Y_{i}$ and a session key sk. $S_{j}$ then computes RM, $V_{s}$ as:
  $$K_{SU}=r_{x}\times X_{a}^{*}\;Y_{i}=r_{s}\times P\;sk=h(AID_{i}||K_{SU}||T_{s})\;RM=\text{Response to the query of }U_{i}\;V_{s}=h(AID_{i}\left|\right|X_{a}\left|\right|Y_{i}\left||RM|\right|T_{s}),$$
  where $T_{s}$ is current timestamp. $S_{j}$ then sends $M_{3}=\langle RM$, $Y_{i}$, $V_{s}$, $T_{s}\rangle$ to A.
8. After receiving $M_{3}$, A retrieves $T_{s}$, and checks the sameness of $V_{s}$. Then, A computes $K_{US}$ and sk as:
  $$V_{s}^{*}=h(AID_{i}\left|\right|X_{a}\left|\right|Y_{i}\left||RM|\right|T_{s})\;verifies\;V_{s}^{*}\overset{?}{=}V_{s}\;K_{US}=r_{a}\times Y_{i}\;sk=h(AID_{i}||K_{US}||T_{s}).$$
  Lastly, A and $S_{j}$ “successfully” agree on a session key sk. Unfortunately, the sensor $S_{j}$ mistakenly believes that he/she is communicating with the legitimate user $U_{i}$.

### 4.2. Sensor Node Impersonation Attack

Park et al. [14] claimed that if A wants to masquerade as the sensor node $S_{j}$, A is required to compute $h(SID_{j}||y)$. This is because the symmetric key $k_{g}=h(h(SID_{j}\left||y)|\right|T_{g})$. However, if A obtains the login request message $M_{1}=$ $\langle AID_{i}$, $X_{i}$, $C_{i}$, $T_{i}$, $W_{i}\rangle$ of the legitimate user $U_{i}$, A then can easily impersonate the sensor node $S_{j}$.

1. After receiving $M_{2}$ from GW, A generates a random number $r_{a}$ and computes $K_{AU}$, $Y_{a}$, RM, $V_{a}$ and a session key sk as:
  $$K_{AU}=r_{a}\times X_{i}\;Y_{a}=r_{a}\times P\;sk=h(AID_{i}||K_{AU}||T_{a})\;RM=\text{Any response to the query of }U_{i}\;V_{a}=h(AID_{i}\left|\right|X_{i}\left|\right|Y_{a}\left||RM|\right|T_{a}),$$
  where $T_{a}$ is current timestamp. A then sends $M_{3}=\langle RM$, $Y_{a}$, $V_{a}$, $T_{a}\rangle$ to $U_{i}$.
2. Upon receiving $M_{3}$ from A, $U_{i}$ retrieves $T_{a}$ and checks the sameness of $V_{a}$. Then, $U_{i}$ computes $K_{UA}$ and sk as:
  $$V_{a}^{*}=h(AID_{i}\left|\right|X_{i}\left|\right|Y_{a}\left||RM|\right|T_{a})\;verifies\;V_{a}^{*}\overset{?}{=}V_{a}\;K_{UA}=r_{i}\times Y_{a}\;sk=h(AID_{i}||K_{UA}||T_{a}).$$
  Lastly, $U_{i}$ and A “successfully” agree on a session key sk. Unfortunately, the user $U_{i}$ mistakenly believes that he/she is communicating with the legitimate sensor $S_{j}$.

### 4.3. Illegal Smart Card Revocation/Reissue Attack

Park et al. [14] claimed that, although A could get the identity $ID_{i}$ in some way, GW checks the legitimacy of the user on the requested identity, and A cannot compute $M_{i}$ and the revocation/reissue request message $Z_{i}$ without the biometric information of $U_{i}$. However, A can modify the revocation/reissue request message. This is because GW cannot distinguish whether or not the user who wishes to revoke $ID_{i}$ is the real user $U_{i}$.

1. Suppose A owns a smart card to extract information $\langle V_{a}$, $N_{a}$, $C_{a}$, h(·), $P_{a}\rangle$ and obtains the identity $ID_{i}$ of the legitimate user $U_{i}$ by using a user impersonation attack.
2. Next, A imprints the personal biometric information $B_{a}$ at the sensor. The sensor hence sketches $B_{a}$ and extracts $\langle R_{a}$, $P_{a}\rangle$ from $Gen\left(B_{a}\right)\to \langle R_{a}$, $P_{a}\rangle$.
3. A computes $A_{a}^{*}=h\left(R_{a}\right)$ and $Z_{i}^{\prime }=ID_{i}⊕N_{a}⊕A_{a}^{*}$ and sends the revocation/reissue request message $\langle ID_{i}$, $ID_{i}^{*}$, $A_{a}^{*}$, $Z_{i}^{\prime }\rangle$ to GW.
4. GW computes $M_{i}^{*}$, $Z_{i}^{*}$, and checks the legitimacy of the user as:
  $$M_{a}^{*}=h(x||y||A_{a}^{*})\;Z_{i}^{*}=ID_{i}⊕M_{a}^{*}\;verifies\;Z_{i}^{\prime }\overset{?}{=}Z_{i}^{*}.$$
5. If this holds, GW revokes $ID_{i}$ and records it on the revocation look-up table. Then, GW computes new authentication parameters $V_{i}$, $N_{i}$ and $C_{i}$ as:
  $$V_{i}=h(ID_{i}^{*}\left|\right|A_{a}^{*})\;N_{i}=M_{a}^{*}⊕A_{a}^{*}\;C_{i}=E_{x}(A_{a}^{*}||up_{i}^{*}).$$
6. GW stores the new authentication parameters $\langle V_{i}$, $N_{i}$, $C_{i}$, h(·)〉 in the smart card $SC_{i}$. GW then reissues $SC_{i}$ to A through a secure channel.
7. A stores $P_{a}$ in the smart card.

Adversary A can revoke the smart card of an authenticated user who does not wish the said smart card to be revoked without permission because GW has no proper process for checking the legitimacy of the user on the previous identity $ID_{i}$.

## 5. The Proposed Scheme

In this section, we will propose a new biometric-based password authentication scheme using a smart card. In our scheme, there are also three participants, the user $U_{i}$, the gateway GW and the sensor $S_{j}$. The GW generates two master keys *x* and *y*, and sends a long-term secret key $h(SID_{j}||y)$ to the sensor $S_{j}$ before the scheme is executed. After that, GW computes x×P where xP is the public key of the gateway. The proposed scheme consists of three phases: registration, login and authentication, and revocation/reissue.

### 5.1. Registration Phase

In this phase, a user $U_{i}$ chooses an identity $ID_{i}$, imprints biometric template $B_{i}$ at the sensor, and then performs the following steps:

1. The sensor sketches $B_{i}$, extracts $\langle R_{i}$, $P_{i}\rangle$ from $Gen\left(B_{i}\right)\to \langle R_{i}$, $P_{i}\rangle$, and stores $P_{i}$ in the memory. $U_{i}$ then sends $\langle ID_{i}$, $A_{i}=h\left(R_{i}\right)\rangle$ to GW over a secure channel.
2. When receiving the registration request message $\langle ID_{i}$, $A_{i}\rangle$ from $U_{i}$, the gateway GW computes the authentication parameters as:
  $$C_{i}=h(ID_{i}\left||x||y\right)\;M_{i}=h\left(C_{i}\right)⊕A_{i}\;N_{i}=x⊕C_{i}⊕y\;V_{i}=h(ID_{i}\left|\right|A_{i}).$$
3. GW stores the authentication parameters $\langle M_{i}$, $N_{i}$, $V_{i}$, h(·)〉 in the smart card $SC_{i}$. GW hence issues $SC_{i}$ to $U_{i}$ via a secure channel.
4. Lastly, $U_{i}$ stores $P_{i}$ in the smart card.

**Figure 1:**
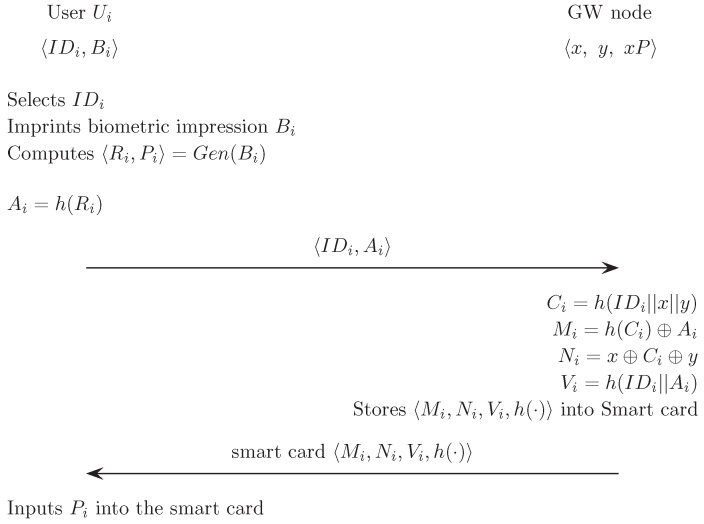
Registration phase of the proposed scheme.

Figure 1 illustrates the registration phase of the proposed scheme.

### 5.2. Login and Authentication Phase

In this phase, $U_{i}$ performs the login phase, and hence $U_{i}$, GW and $S_{j}$ verify each other’s authenticity. Finally, $U_{i}$ and $S_{j}$ generates a common session key in this phase as follows:

1. $U_{i}$ inserts his/her smart card $SC_{i}$ into the card reader, inputs the identity $ID_{i}$, and imprints the personal biometrics $B_{i}^{*}$ at the sensor.
2. The sensor then sketches $B_{i}^{*}$ and extracts $R_{i}$ from $Rep(B_{i}^{*}$, $P_{i})\to \langle R_{i}\rangle$. Then, $SC_{i}$ computes $A_{i}^{*}$ and $V_{i}^{*}$ using fuzzy extraction and compares $V_{i}^{*}$ with $V_{i}$ as:
  $$R_{i}^{*}=Rep\left(B_{i}^{*},P_{i}\right)\;A_{i}^{*}=h\left(R_{i}^{*}\right)\;V_{i}^{*}=h(ID_{i}\left|\right|A_{i}^{*})\;verifies\;V_{i}\overset{?}{=}V_{i}^{*}.$$
3. $SC_{i}$ generates random numbers $r_{1}$ and $r_{2}$ and hence computes
  $$X_{i}=r_{1}\times P\;h\left(C_{i}\right)=M_{i}⊕A_{i}^{*}\;AID_{i}=ID_{i}⊕h\left(r_{2}\right)\;M_{1}=r_{2}⊕h\left(C_{i}\right)\;M_{2}=h(AID_{i}||h\left(C_{i}\right)\left|\right|X_{i}\left|\right|r_{2}\left|\right|T_{i})\;M_{3}=N_{i}⊕\left(r_{1}\times xP\right),$$
  where $T_{i}$ is current timestamp. Then, $U_{i}$ sends the login request message $\langle AID_{i}$, $X_{i}$, $M_{1}$, $M_{2}$, $M_{3}$, $T_{i}\rangle$ to GW.
4. Upon receiving a login request message from $U_{i}$, GW retrieves $T^{\prime }$ and verifies $T^{\prime }-T_{i}\leq △T$. If this is true, GW computes $C_{i}^{*}$, $r_{2}^{*}$, $ID_{i}^{*}$ and $M_{2}^{*}$ and compares $C_{i}^{*}$ with $h(ID_{i}^{*}||x||y)$ and $M_{2}^{*}$ with $M_{2}$ as:
  $$C_{i}^{*}=M_{3}⊕\left(x\times X_{i}\right)⊕x⊕y\;r_{2}^{*}=M_{1}⊕h\left(C_{i}^{*}\right)\;ID_{i}^{*}=AID_{i}⊕h\left(r_{2}^{*}\right)\;verifies\;C_{i}^{*}=h(ID_{i}^{*}\left||x||y\right)\;M_{2}^{*}=h(AID_{i}||h\left(C_{i}^{*}\right)\left|\right|X_{i}\left|\right|r_{2}^{*}\left|\right|T_{i})\;verifies\;M_{2}\overset{?}{=}M_{2}^{*}.$$
  If this holds, GW verifies the legitimacy of $U_{i}$.
5. GW computes $k_{g}$, $C_{g}$ and $W_{g}$,
  $$k_{g}=h(h(SID_{j}\left||y)|\right|T_{g})\;C_{g}=E_{k_{g}}(AID_{i}\left|\right|r_{2}\left|\right|X_{i})\;W_{g}=h(h(SID_{j}||y)||AID_{i}\left|\right|C_{g}\left|\right|T_{g}),$$
  where $T_{g}$ is current timestamp. GW then sends the authentication message $\langle AID_{i}$, $C_{g}$, $T_{g}$, $W_{g}\rangle$ to $S_{j}$.
6. When receiving the authentication message from GW, $S_{j}$ retrieves $T^{\prime \prime }$ and verifies $T^{\prime \prime }-T_{g}\leq △T$. If this is true, $S_{j}$ verifies the validity of $W_{g}$ by comparing it with $h(h(SID_{j}||y)||AID_{i}\left|\right|C_{g}\left|\right|T_{g})$ to check the legitimacy of GW. After that, $S_{j}$ computes $k_{g}^{*}$, and decrypts $C_{g}$ using $k_{g}^{*}$. Then, $S_{j}$ checks the validity of the received $AID_{i}$ by comparing the computed $AID_{i}^{*}$ as
  $$k_{g}^{*}=h(h(SID_{j}\left||y)|\right|T_{g})\;D_{k_{g}^{*}}=AID_{i}^{*}\left|\right|r_{2}^{*}\left|\right|X_{i}^{*}\;verifies\;AID_{i}\overset{?}{=}AID_{i}^{*}.$$
7. $S_{j}$ generates a random number $r_{s}$ and computes $K_{SU}$, $Y_{i}$, RM, $V_{s}$ and a session key sk as:
  $$K_{SU}=r_{s}\times X_{i}^{*}\;Y_{i}=r_{s}\times P\;sk=h(AID_{i}||K_{SU}||T_{s})\;RM=\text{Response to the query of }U_{i}\;V_{s}=h(AID_{i}\left|\right|r_{2}^{*}\left|\right|Y_{i}\left||sk||RM|\right|T_{s}),$$
  where $T_{s}$ is current timestamp. $S_{j}$ then sends 〈RM, $Y_{i}$, $V_{s}$, $T_{s}\rangle$ to $U_{i}$.
8. After receiving response message 〈RM, $Y_{i}$, $V_{s}$, $T_{s}\rangle$ from $S_{j}$, $U_{i}$ computes sk and checks whether $V_{s}^{*}$ is equal to $V_{s}$:
  $$K_{US}=r_{1}\times Y_{i}\;sk=h(AID_{i}||K_{US}||T_{s})\;V_{s}^{*}=h(AID_{i}\left|\right|r_{2}\left|\right|Y_{i}\left||sk||RM|\right|T_{s})\;verifies\;V_{s}^{*}\overset{?}{=}V_{s}.$$
  The legitimate user $U_{i}$ can only compute $K_{US}$ and sk. $U_{i}$ and $S_{j}$ can communicate securely using the common session key sk.

**Figure 2:**
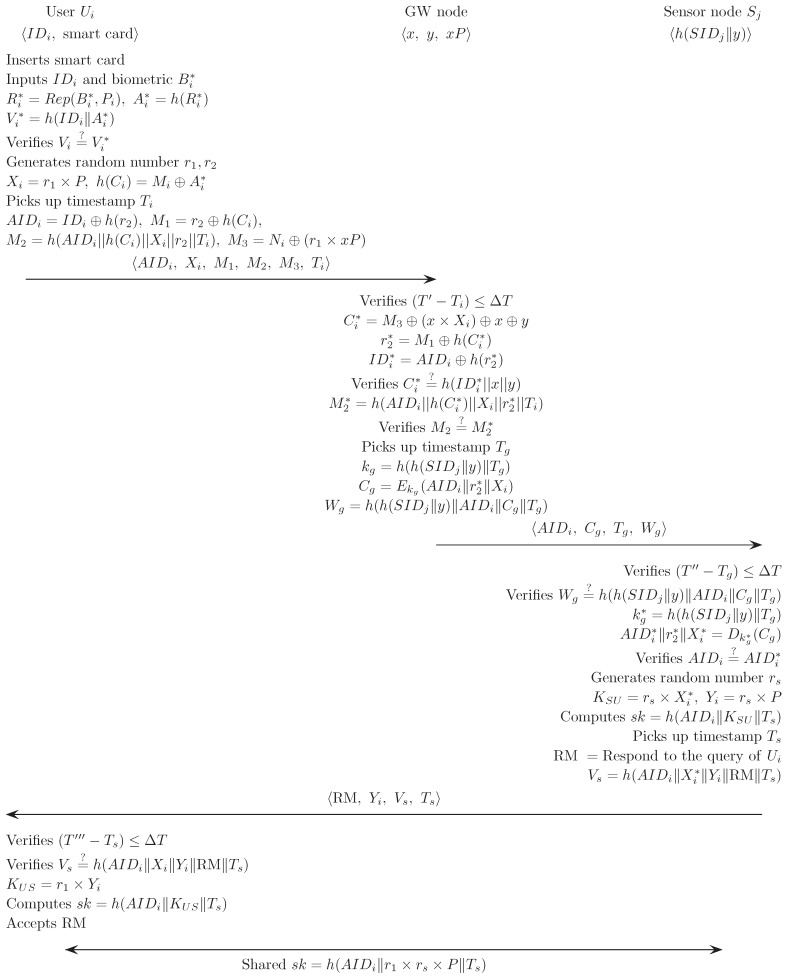
Login and authentication phase of the proposed scheme.

Figure 2 illustrates the login and authentication phase of the proposed scheme.

### 5.3. Revocation or Reissue Phase

To make up for smart card loss or long term key disclosure, the smart card should be revoked and reissued in cycles.

1. If $U_{i}$ wants to revoke and reissue a smart card, he/she inserts his/her smart card $SC_{i}$ into the card reader, inputs the previous identity $ID_{i}$ and the new identity $ID_{i}^{*}$ to prevent adversaries from registering with the same identity $ID_{i}$, and then imprints the personal biometrics $B_{i}^{*}$ at the sensor.
2. The sensor then sketches $B_{i}^{*}$ and extracts $R_{i}$ from $Rep(B_{i}^{*}$, $P_{i})\to \langle R_{i}\rangle$. Then, $SC_{i}$ computes $A_{i}^{*}$ and $V_{i}^{*}$ using fuzzy extraction,
  $$R_{i}=Rep\left(B_{i}^{*},P_{i}\right)\;A_{i}=h\left(R_{i}\right).$$
3. $U_{i}$ computes $Z_{i}=ID_{i}⊕M_{i}$ and sends the revocation/reissue request message $\langle ID_{i}$, $ID_{i}^{*}$, $A_{i}$, $Z_{i}\rangle$ to GW over a secure channel.
4. GW first checks whether $ID_{i}$ is the same as $ID_{i}^{*}$ or not. If they are different, GW computes $M_{i}^{*}$, $Z_{i}^{*}$ and checks the legitimacy of the user as:
  $$C_{i}^{*}=h(ID_{i}\left||x||y\right)\;Z_{i}^{*}=ID_{i}⊕h\left(C_{i}^{*}\right)⊕A_{i}\;verifies\;Z_{i}^{*}\overset{?}{=}Z_{i}.$$
5. If this is true, GW revokes $ID_{i}$ and records it on the revocation look-up table. Then, GW computes new authentication parameters $V_{i}$, $N_{i}$ and $C_{i}$ as:
  $$C_{i}=h(ID_{i}^{*}\left||x||y\right)\;M_{i}=h\left(C_{i}\right)⊕A_{i}\;N_{i}=x⊕C_{i}⊕y\;V_{i}=h(ID_{i}^{*}\left|\right|A_{i}).$$
6. GW stores h(·) and the new authentication parameters $\langle M_{i}$, $N_{i}$, $V_{i}$, h(·)〉 in the smart card $SC_{i}$. GW then reissues $SC_{i}$ to $U_{i}$ through a secure channel.
7. $U_{i}$ stores $P_{i}$ in the smart card.

**Figure 3:**
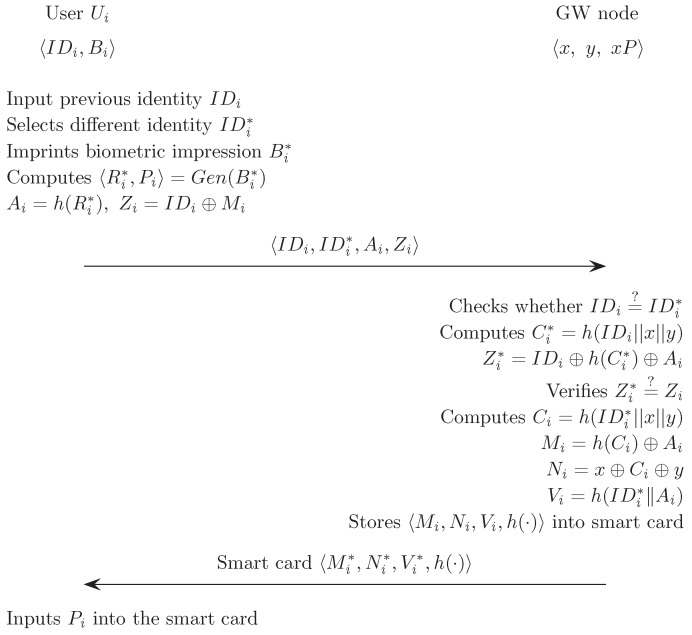
Revocation/reissue phase of proposed scheme.

Figure 3 illustrates the revocation/reissue phase of the proposed scheme.

## 6. Security Analysis

In this section, we demonstrate that the proposed scheme, which retains the merits of Park et al.’s scheme [14], can withstand several types of possible attacks; and we also show that the scheme supports several security properties. The security analysis of the proposed scheme was conducted with the threat assumptions made in the Preliminaries.

### 6.1. Formal Security Analysis

In this subsection, we have demonstrated that the proposed scheme is secure through a formal proof using the random oracle model [18,25]. At first, we specify a collision-free one-way hash function as follows.

**Definition** **6.** The collision-resistance one-way hash function f:{0, ${1\}}^{*}\to \{0$, ${1\}}^{n}$ pick up an input as a binary string x∈{0, ${1\}}^{*}$ that has a random length, produces a binary string h(x)∈{0, ${1\}}^{n}$, and gratifies the following requirements:

- Given the y∈Y, it is not possible to find out computationally about x∈X such that y=h(x).
- Given the x∈X, it is not possible to find out computationally about another $x^{\prime }\neq x\in X$, such that $h\left(x^{\prime }\right)=h\left(x\right)$.
- It is not possible to find out computationally about a pair $(x^{\prime }$, $x)\in X^{\prime }\times X$, with $x^{\prime }\neq x$, such that $h\left(x^{\prime }\right)=h\left(x\right)$.

**Theorem** **1.** *According to the assumption that if the collision-free one-way hash function h(·) closely acts like an oracle, the proposed scheme is then distinctly secure against an adversary A for the protection of the sensitive information including the identity $ID_{i}$, nearly random binary string $r_{2}$ and master secret key x, y of the gateway node GW.*

**Proof.**  This random oracle can extract the input value *x* from the given hash result y=h(x) without fail. A now executes the experimental algorithm as shown in Algorithm 1, $EXP_{HASH,\;A}^{JHKAS},$ for the proposed scheme as JHKAS, for example. Let us define the probability of success for $EXP_{HASH,A}^{JHKAS}$ as $Success_{HASH,\;A}^{JHKAS}=\left|Pr\left[EXP_{HASH,\;A}^{JHKAS}=1\right]-1\right|$, where Pr(·) means the probability of $EXP_{HASH,A}^{JHKAS}$. The advantage function for this algorithm then becomes $Adv_{HASH,\;A}^{JHKAS}\left(t,\;q_{R}\right)=max_{Success}$, where the *t* is the execution time and $q_{R}$ is the number of queries. Discuss the algorithm as shown in Algorithm 1 for the A. If A has the capability to solve the hash function problem given in Definition 6, then he/she can immediately retrieve the identity $ID_{i}$, nearly random binary string $r_{2}$ and master secret key *x*, *y* of the gateway node GW. In that case, the A will detect the complete connections between the $U_{i}$ and the GW; however, the inversion of the input from a given hash value is not possible computationally, i.e., $Adv_{HASH,\;A}^{JHKAS}\left(t\right)\leq \epsilon$, for all ϵ>0. Thus, $Adv_{HASH,\;A}^{JHKAS}\left(t,\;q_{R}\right)\leq \epsilon$, since $Adv_{HASH,\;A}^{JHKAS}\left(t,\;q_{R}\right)$ depends on $Adv_{HASH,\;A}^{JHKAS}\left(t\right)$. In conclusion, there is no way for A to detect the complete connections between the $U_{i}$ and the GW, and the proposed scheme is distinctly secure to an adversary A for retrieving ($ID_{i}$, $r_{2}$, *x*, *y*). ☐

**Algorithm 1**
$EXP_{HASH,\;A}^{JHKAS}$1.  Eavesdrop login request message $\langle AID_{i}$, $X_{i}$, $M_{1}$, $M_{2}$, $M_{3}$, $T_{i}\rangle$ during the login phase. 2.  Call the Reveal oracle. Let $(AID_{i}^{\prime }$, $h{\left(C_{i}\right)}^{\prime }$, $X_{i}^{\prime }$, $r_{2}^{\prime }$, $T_{i}^{\prime })\leftarrow$ Reveal($M_{2}$) 3.  **if** ($AID_{i}^{\prime }$ == $AID_{i}$) **then** 4.   Accept $h{\left(C_{i}\right)}^{\prime }$, $X_{i}^{\prime }$, $r_{2}^{\prime }$, $T_{i}^{\prime }$ as the correct of user $U_{i}$ 5.   Call the Reveal oracle. Let ($C_{i}^{\prime })\leftarrow$ Reveal($h{\left(C_{i}\right)}^{\prime }$) 6.   Call the Reveal oracle. Let ($C_{i}^{\prime \prime })\leftarrow$ Reveal($M_{1}⊕r_{2}$) 7.   **if** ($C_{i}^{\prime }$ == $C_{i}^{\prime \prime }$) **then** 8.    Accept the $C_{i}$ as the correct of user $U_{i}$ 9.    Call the Reveal oracle. Let $(ID_{i}^{\prime }$, $x^{\prime }$, $y^{\prime })\leftarrow$ Reveal($C_{i}$) 10.   Compute $ID_{i}^{\prime \prime }=AID_{i}⊕h\left(r_{2}\right)$ 11.   **if** ($ID_{i}$ == $ID_{i}^{\prime \prime }$) **then** 12.    Accept $x^{\prime }$, $y^{\prime }$ as the correct secret key *x*, *y* of gateway node GW 13.    **return 1(Success)** 14.   **else** 15.    **return 0** 16.  **else** 17.   **return 0** 18.  **end if** 19. **else** 20.  **return 0** 21. **end if**

### 6.2. Simulation for Formal Security Verification Using the AVISPA Tool

In this subsection, we simulate the proposed scheme using the widely accepted AVISPA for the formal security verification. The main purpose of the simulation is to verify whether the proposed scheme is secure to replay and man-in-the middle attacks. The AVISPA tool consists of four back-ends: (i) On-the-fly Model-Checker (OFMC); (ii) Constraint-Logic-based Attack Searcher; (iii) SAT-based Model Checker; and (iv) Tree Automata based on Automatic Approximations for the Analysis of Security Protocols. In the AVISPA, the protocol is implemented in HLPSL [26], which is based on: the basic roles for representing each participant role and composition roles for representing the scenarios of the basic roles. The basic types available in the HLPSL are [27]:

- agent : The agent denotes a principal name. The intruder always has the special identifier *i*.
- symmetric_key : The symmetric_key is the key for a symmetric-key cryptosystem.
- text : The text values are often used as nonces. They can also be applied for messages.
- nat : The nat is used for denoting the natural numbers in non-message contexts.
- const : This type represents constants.
- hash_func : The base type hash_func represents cryptographic one-way hash functions.

The role of the initiator, the user $U_{i},$ is provided in Algorithm 2. The $U_{i}$ first receives the start signal and updates its state value from 0 to 1. The state value is maintained by the variable *State*. In a similar way, the roles of the gateway GW and sensor node $S_{j}$ of the proposed scheme are implemented and shown in Algorithm 3 and 4, respectively. The specifications in HLPSL language for the role of session, goal, and environment are specified in Algorithm 5. The simulation result for the formal security verification of the proposed scheme using CL-AtSe is shown in Algorithm 6. It is clear that the proposed scheme is secure to passive and active attacks including the replay and man-in-the middle attacks.

**Algorithm 2** Role specification for user $U_{i}$role user (Ui, GW, Sj: agent, SKug, SKus: symmetric_key, H, F: function, SND, RCV: channel (dy)) played_by Ui def= local State : nat, IDi, Ri, P, Ai, Mi, Ni, Vi: text, AIDi, R1, R2, Xi, Ci, Di, M1, M2, M3, Ti, Rs: text, RM, Yi, Vs, Ts, Gx, Gy, Kus: text init State := 0 transition 0. State = 0 ∧ RCV(start) =|> State’:= 5 ∧ Ai’ := H(Ri) ∧ secret(Ri, scrt0, Ui) ∧ secret(IDi, scrt1, {Ui, GW}) ∧ SND({IDi.Ai’}_SKug) 5. State = 2 ∧ RCV({Mi’.Ni’.Vi’}_SKug.P’) =|> State’:= 8 ∧ R1’:=new() ∧ R2’:=new() ∧ Ti’:=new() ∧ Xi’:=F(R1’.P’) ∧ Di’:=xor(Mi’, Ai’) ∧ AIDi’:=xor(IDi, H(R2’)) ∧ M1’:=xor(R2’, Di’) ∧ M2’:=H(AIDi’.Di’.Xi’.R2’.Ti’) ∧ M3’:=xor(Ni’, F(R1’.F(Gx’.P))) ∧ secret({Gx’, Gy’}, scrt2, GW) ∧ SND(AIDi’, Xi’, M1’, M2’, M3’, Ti’) ∧ witness(Ui, Sj, ui_sj_r1, R1’) 8. State = 8 ∧ RCV({RM’.Yi’.Vs’.Ts’}_SKug.P’) =|> State’:= 9 ∧ Vs’:=H(AIDi’.Xi’.Yi’.RM’.Ts’) ∧ Kus’:= F(R1’.F(Rs’.P)) ∧ SKus’:=H(AIDi’.Kus’.Ts’) ∧ witness(Ui, GW, ui_gw_r2, R2’) ∧ request(Sj, Ui, sj_ui_rs, Rs’) end role

**Algorithm 3** Role specification for gateway GWrole gateway (Ui, GW, Sj: agent, SKug, SKgs, Kg: symmetric_key, H, F: function, SND, RCV: channel (dy)) played_by GW def= local State : nat, IDi, R1, R2, Ri, P, Ai, Mi, Ni, Vi, Rs: text, AIDi, SIDj, Ks, Xi, Ci, Di, M1, M2, M3, Ti: text, Cg, Tg, Wg, Gx, Gy: text init State := 1 transition 1. State = 1 ∧ RCV({IDi.Ai’}_SKug) =|> State’:= 6 ∧ Ci’:= H(IDi.Gx’.Gy’) ∧ P’:=new() ∧ Di’:=H(Ci’) ∧ Mi’:=xor(Di’, H(Ri)) ∧ Ni’:=xor(Gx’, Ci’, Gy’) ∧ Vi’:=H(IDi.H(Ri)) ∧ secret({Gx’, Gy’}, scrt2, GW) ∧ SND({Mi’.Ni’.Vi’}_SKug.P’) 3. State = 3 ∧ RCV({SIDj}_SKgs) =|> State’:= 6 ∧ Ks’:=H(SIDj.Gy’) ∧ SND({Ks’}_SKgs.P’) 6. State = 6 ∧ RCV(AIDi’.Xi’.M1’.M2’.M3’.Ti’) =|> State’:= 9 ∧ Tg’ = new() ∧ Di’:=H(xor(M3’, F(R1’.F(Gx’.P)), Gx’, Gy’)) ∧ R2’:=xor(M1’, Di’) ∧ IDi’:=xor(AIDi’, H(R2’)) ∧ Kg’:=H(H(SIDj’.Gy’).Tg’) ∧ Cg’:={AIDi’.R2’.Xi’}_Kg’ ∧ Wg’:=H(H(SIDj’.Gy’).AIDi’.Cg’.Tg’) ∧ secret({Gx’, Gy’}, scrt2, GW) ∧ secret(H(SIDj’.Gy’), scrt4, {GW, Sj}) ∧ SND(AIDi’.Cg’.Tg’.Wg’) ∧ request(Ui, Sj, ui_sj_r1, R1’) end role

**Algorithm 4** Role specification for sensor $S_{j}$role sensor (Ui, GW, Sj: agent, SKgs, Kg, SKus: symmetric_key, H, F: function, SND, RCV: channel (dy)) played_by Sj def= local State : nat, IDi, Ri, P, Ai, Mi, Ni, Vi, R1, R2: text, AIDi, SIDj, Ks, Xi, Ci, M1, M2, M3, Ti: text, Cg, Tg, Wg: text, RM, Yi, Vs, Ts, Gx, Gy, Rs, Kus:text init State := 2 transition 2. State = 2 ∧ RCV(start) =|> State’:= 4 ∧ SND({SIDj}_SKgs.P’) 4. State = 4 ∧ RCV({H(SIDj.Gy’)}_SKgs.P’) =|> State’:= 7 ∧ secret({Gx’, Gy’}, scrt2, GW) 7. State = 7 ∧ RCV(AIDi’.Xi’.M1’.M2’.M3’.Ti’) =|> State’:= 10∧ Kg’:=H(H(SIDj’.Gy’).Tg’) ∧ Kus’:=F(R1’.F(Rs’.P)) ∧ Yi’:=F(Rs’.P) ∧ Ts’:=new() ∧ SKus’:=H(AIDi’.Kus’.Ts’) ∧ RM’:=new() ∧ Vs’:=H(AIDi’.Xi’.Yi’.RM’.Ts’) ∧ SND(RM’, Yi’, Vs’, Ts’) ∧ secret(H(SIDj.Gy’), scrt4, {GW, Sj}) ∧ witness(Sj, Ui, sj_ui_rs, Rs’) ∧ request(Ui, Sj, ui_sj_r1, R1’) end role

**Algorithm 5** Role specification for session, goal and environmentrole session(Ui, GW, Sj: agent, SKug, SKus, SKgs, Kg: symmetric_key, H, F: function) def= local Z1, Z2, Z3, S1, S2, S3: channel (dy) composition user(Ui, GW, Sj, SKug, SKus, H, F, Z1, S1) ∧ gateway(Ui, GW, Sj, SKug, SKgs, Kg, H, F, Z2, S2) ∧ sensor(Ui, GW, Sj, SKgs, Kg, SKus, H, F, Z3, S3) end role role environment() def= const ui, gw, sj : agent, skug, skgs, skus, kg : symmetric_key, h, f : function, aidi, sidj, p, xi : text, xi, m1, m2, m3, ti : text, cg, tg, wg : text, rm, yi, vs, ts : text, ui_sj_r1, ui_gw_r2, sj_ui_rs : protocol_id, scrt0, scrt1, scrt2, scrt3, scrt4 : protocol_id intruder_knowledge = {ui, gw, sj, h, f, p, aidi, sidj, xi, m1, m2, m3, ti, cg, tg, wg, rm, yi, vs, ts} composition session(ui, gw, sj, skug, skgs, kg, skus, h, f) end role goal secrecy_of scrt0 secrecy_of scrt1 secrecy_of scrt2 secrecy_of scrt3 secrecy_of scrt4 authentication_on ui_sj_r1 authentication_on ui_gw_r2 authentication_on sj_ui_rs end goal

**Algorithm 6** Role specification for session, goal and environmentSUMMARY SAFE DETAILS BOUNDED_NUMBER_OF_SESSIONS PROTOCOL /home/span/span/testsuite/results/testrv.if GOAL As Specified BACKEND CL-AtSe STATISTICS Analysed : 1 states Reachable : 0 states Translations: 0.03 s Computation: 0.00 s

### 6.3. Informal Security Analysis

Table 2 compares the security features provided by the proposed scheme with other related schemes.

#### 6.3.1. User Anonymity

Suppose an adversary A intercepts the login request message $\langle AID_{i}$, $X_{i}$, $M_{1}$, $M_{2}$, $M_{3}$, $T_{i}\rangle$ of a legitimate user $U_{i}$. However, $ID_{i}$ cannot be derived from $AID_{i}$ without the knowledge of a random number $r_{2}$; furthermore, the $r_{2}$ cannot be derived from $M_{1}$ without a hash value $h\left(C_{i}\right)=h(h(ID_{i}\left||x||y)\right)$. The $U_{i}$ and GW can only compute $h(C_{i}$). The proposed scheme therefore provides user anonymity.

#### 6.3.2. Mutual Authentication

The proposed scheme not only guarantees secrecy as the other authentication scheme, but also $U_{i}$, $S_{j}$ and GW authenticate each other. GW authenticates $U_{i}$ by checking whether $M_{2}$ is valid or not because only a legitimate user can compute a valid $h(C_{i})$ using a biometric template. Then, $S_{j}$ authenticates GW by checking $W_{g}$, which only GW can compute using the shared long-term key $h(SID_{j}||y)$ and the time stamp $T_{g}$. Finally, the $U_{i}$ authenticates $S_{j}$ by checking the validity of $V_{s}$ because only $U_{i}$ and $S_{j}$ can compute the session key sk.

#### 6.3.3. Message Confidentiality

Message confidentiality is an important security aspect that provides secrecy by limiting the adversary’s access to the message. Communication messages in the public channel do not affect the disclosure of secret values, such as $ID_{i}$, $r_{1}$, $r_{2}$, $r_{s}$ and sk. A cannot compute important information from $AID_{i}$, $X_{i}$, $M_{1}$, $M_{2}$, $M_{3}$, $C_{g}$, $W_{g}$, $Y_{i}$ and $V_{s}$. Furthermore, $T_{i}$, $T_{g}$, $T_{s}$ and RM are basically public information, so they do not need to be protected.

#### 6.3.4. Perfect Forward Secrecy

The perfect forward secrecy means that if one long-term key is compromised, a session key that is derived from these long-term keys will not be compromised in the future [28]. In our scheme, a session key sk between user $U_{i}$ and sensor $S_{j}$ is calculated as follows:

$$X_{i}=r_{1}\times P\;Y_{i}=r_{s}\times P\;K_{US}=K_{SU}=r_{s}\times X_{i}=r_{1}\times Y_{i}\;sk=h(AID_{i}||K_{US}||T_{s}).$$

Even if the gateway GW’s long-term key (*x*, *y*) is compromised, adversary A cannot retrieve $r_{1}$ and $r_{s}$ to generate the session keys between $U_{i}$ and $S_{j}$. The session key of our proposed scheme is based on a elliptic curve discrete logarithm problem (ECDLP). An adversary A cannot obtain $r_{1}\times r_{s}\times P$ from $r_{1}\times P$ and $r_{s}\times P$. Our scheme therefore provides the perfect forward secrecy.

#### 6.3.5. User Impersonation Attack

Suppose A owns a smart card to extract information $\langle V_{a}$, $N_{a}$, $C_{a}$, h(·), $P_{a}\rangle$ and intercepts the login request message $\langle AID_{i}$, $X_{i}$, $M_{1}$, $M_{2}$, $M_{3}$, $T_{i}\rangle$ of legitimate user $U_{i}$. A can then try modifying a login request message. Even if A guesses or obtains $U_{i}$’s identity $ID_{i}$, GW verifies whether $C_{i}^{*}$ is equal to $h(ID_{i}^{*}||x||y)$. The A cannot compute $C_{i}$ and $h(C_{i})$, and then fails to impersonate a legitimate user $U_{i}$. The proposed scheme therefore can resist user impersonation attack.

#### 6.3.6. Gateway or Sensor Node Impersonation Attack

If A wants to masquerade as the gateway node GW or a sensor node $S_{j}$, the hash value $h(SID_{j}||y)$ is needed. However, it is computationally difficult to guess $h(SID_{j}||y)$ or $k_{g}$ correctly. Furthermore, even if A obtains the login request message $\langle AID_{i}$, $X_{i}$, $M_{1}$, $M_{2}$, $M_{3}$, $T_{i}\rangle$, A does not know $r_{2}$. Thus, $V_{s}=h(AID_{i}\left|\right|r_{2}\left|\right|Y_{i}\left||sk||RM|\right|T_{s})$ cannot be computed. The proposed scheme therefore can resist gateway or sensor node impersonation attack.

#### 6.3.7. Illegal Smart Card Revocation/Reissue Attack

Even if A obtains the identity $ID_{i}$ of a legitimate user $U_{i}$, A cannot compute $h(C_{i})$ without the value $A_{i}=h\left(R_{i}\right)$. Furthermore, GW checks the legitimacy of the user on the request identity by computing $C_{i}^{*}=h(ID_{i}\left||x||y\right)$ and $Z_{i}^{*}=ID_{i}⊕h\left(C_{i}^{*}\right)⊕A_{i}$. Therefore, even if A sends the revocation/reissue request message $\langle ID_{i}$, $ID_{i}^{*}$, $A_{a}=h\left(R_{a}\right)$, $Z_{a}=ID_{i}⊕h\left(C_{a}\right)⊕A_{a}\rangle$ to GW, A fails to revoke $ID_{i}$ and reissue the smart card with $ID_{i}$. The proposed scheme therefore can resist an illegal smart card revocation/reissue attack.

#### 6.3.8. Session Key Exposure by GW

The gateway GW can intercept communication messages and obtain both $X_{i}=r_{1}\times P$ and $Y_{i}=r_{s}\times P$. However, GW cannot derive $r_{1}$ and $r_{s}$ and therefore cannot compute the common session key sk. This is because our proposed scheme based on the elliptic curve discrete logarithm problem (ECDLP).

#### 6.3.9. Denial of Service Attack

In the proposed scheme, $U_{i}$, $S_{j}$ and GW basically check for freshness of timestamp in each authentication step. Each message for verification such as $M_{2}$, $W_{g}$ and $V_{s}$ includes the current timestamp *T*. Furthermore, each entity checks whether the calculated value is equal to the received value. The proposed scheme can resist denial of service attack.

#### 6.3.10. User Verification Problem

GW checks for the sameness in the identity $ID_{i}$ to verify the status a legitimate user $U_{i}$ by computing $C_{i}^{*}=h(ID_{i}^{*}\left||x||y\right)$. Furthermore, $U_{i}$ can compute constant values including $A_{i}=h\left(R_{i}\right)$ as a result of the fuzzy extractor. GW can authenticate a legal user even if the user inputs slightly different biometric information $B_{i}^{*}$. Our proposed scheme therefore can prevent user verification problems.

#### 6.3.11. Stolen Verifier Attack

In the proposed scheme, GW and $S_{j}$ do not store any identification, password table or user biometrics. GW stores only the master secret key (*x*, *y*), and $S_{j}$ stores only $h(SID_{j}||y)$. The proposed scheme therefore can resist stolen verifier attacks.

#### 6.3.12. Replay Attack

Even if the adversary A obtains the communication message, and sends them again with the current timestamps $T_{i}$, $T_{g}$, and $T_{s}$, A cannot compute $M_{2}$, $W_{g}$, $V_{s}$ using the current timestamps. The proposed scheme therefore can resist replay attacks.

## 7. Performance Analysis

In this section, we compare the computational costs of the proposed scheme with the other related schemes [13,14,29,30]. Table 3 shows a comparison of the computational costs of the proposed scheme with the other related schemes. In the comparisons, XOR operations are not considered because these also can be ignored. Compared to Park et al.’s scheme [14], the proposed scheme performs three further hash operations and two elliptic curve computations. However, we reduce three encryption/decryption operations. Additionally, the proposed scheme provides the revocation and reissue phase, and can resist well-known attacks.

## 8. Conclusions

The various authentication schemes for WSNs have been proposed. Recently, Park et al. demonstrated the security vulnerabilities of Choi et al.’s scheme and proposed an enhanced authentication scheme. However, in this paper, we have identified vulnerabilities in Park et al.’s scheme in terms of impersonation and revocation/reissue. To overcome these vulnerabilities, we proposed a new biometric-based authentication scheme with improved security. Security and performance analysis shows that our proposed scheme is more secure and efficient than other related schemes.

## Figures and Tables

**Table 1:** Notations used in this paper.

Term	Description
$U_{i}$	user *i*
A	adversary
$B_{i}$	biometric template of $U_{i}$
$E_{k}\left(\cdot \right)/D_{k}\left(\cdot \right)$	encryption or decryption with key *k*
GW	gateway node
$G_{1}$	cyclic groups of order *q*
h(·)	hash function
$h(SID_{j}||y)$	long-term secret of $S_{j}$ generated by GW
$ID_{i}$	actual identity of $U_{i}$
*P*	generator of $G_{1}$
$r_{i}$, $r_{1}$, $r_{2}$	random number generated by $U_{i}$
$r_{s}$	random number generated by $S_{j}$
$S_{j}$	sensor node *j*
$SC_{i}$	smart card of user $U_{i}$
$SID_{j}$	identity of $S_{j}$
$T_{i}$	time stamp
$up_{i}$	*i*-th update phase
x,y	two master keys of GW
RM	Response to the query message
‖	concatenation operation
⊕	bitwise XOR operation

**Table 2:** Comparison of security features.

Features	Yoon and Kim	Choi et al.	Park et al.	The Proposed
	[12]	[13]	[14]		
Provides user anonymity	N/A	×	◯	◯
Provides mutual authentication	◯	◯	◯	◯
Provides message confidentiality	◯	◯	◯	◯
Provides perfect forward secrecy	N/A	◯	◯	◯
Resists insider attack	◯	×	◯	◯
Resists impersonation attack	◯	×	×	◯
Resists illegal smart card revocation/reissue attack	×	×	×	◯
Resists biometric recognition error	×	◯	◯	◯
Resists session key exposure by gateway	×	◯	◯	◯
Resists denial of service attack	×	◯	◯	◯
Resists user verification problem	×	◯	◯	◯
Resists stolen verifier attack	◯	◯	◯	◯
Resists replay attack	◯	◯	◯	◯
Security factor	Two-factor	Two-factor	Two-factor	Two-factor

◯: scheme provides the property; ×: scheme does not provide the property; N/A: scheme does not consider the property.

**Table 3:** Comparison of computational costs.

Phases		Choi et al.	Park et al.	Nam et al.	Park et al.	The Proposed
		[13]	[14]	[29]	[30]	
Registration	$U_{i}$	$T_{H}$ + $T_{F}$	$T_{H}$ + $T_{F}$	$T_{H}$	$T_{H}$ + $T_{F}$	$T_{H}$ + $T_{F}$
	GWN	3$T_{H}$	2$T_{H}$ + $T_{E}$	$T_{H}$ + $T_{E}$ + $T_{e}$	5$T_{H}$	$T_{e}$ + 3$T_{H}$
	$S_{j}$	-	-	-	-	-
Login and authentication	$U_{i}$	$10T_{H}$ + $T_{F}$ + $T_{E}$ + 2$T_{e}$	$6T_{H}$ + $T_{F}$ + 2$T_{e}$	3$T_{H}$ + 3$T_{e}$ + $T_{E}$ + $T_{M}$	10$T_{H}$ + $T_{F}$ + 2$T_{e}$	$6T_{H}$ + $T_{F}$ + 3$T_{e}$
	GWN	10$T_{H}$ + 2$T_{E}$	7$T_{H}$ + 2$T_{E}$	$T_{H}$ + 2$T_{E}$ + $T_{e}$ + 3$T_{M}$	11$T_{H}$	6$T_{H}$ + $T_{e}$ + $T_{E}$
	$S_{j}$	6$T_{H}$ + $T_{E}$ + 2$T_{e}$	4$T_{H}$ + $T_{E}$ + 2$T_{e}$	$T_{H}$ + 2$T_{e}$ + 2$T_{M}$	4$T_{H}$ + 2$T_{e}$	4$T_{H}$ + $T_{E}$ + 2$T_{e}$
Revocation and reissue	$U_{i}$	$T_{H}$ + $T_{F}$	$T_{H}$ + $T_{F}$	-	-	$T_{H}$ + $T_{F}$
	GWN	3$T_{H}$	2$T_{H}$ + $T_{E}$	-	-	5$T_{H}$
	$S_{j}$	-	-	-	-	-
Total cost		34$T_{H}$ + 3$T_{F}$	23$T_{H}$ + 3$T_{F}$	7$T_{H}$ + 4$T_{E}$	31$T_{H}$ + 2$T_{F}$	26$T_{H}$ + 3$T_{F}$
		+ 4$T_{E}$ + 4$T_{e}$	+ 5$T_{E}$ + 4$T_{e}$	+ 7$T_{e}$ + 6$T_{M}$	+ 4$T_{e}$	+ 2$T_{E}$ + 6$T_{e}$

$T_{e}$: computational time for elliptic curve computation; $T_{E}$: computational time for encryption/decryption; $T_{F}$: computational time for fuzzy extraction; $T_{H}$: computational time for hash function; $T_{M}$: computational time for massage authentication code.

## References

[1] Yick J., Mukherjee B., Ghosal D. (2008). Wireless sensor network survey. Comput. Netw..

[2] Watro R., Kong D., Cuti S., Gardiner C., Lynn C., Kruus P. TinyPK: Securing sensor networks with public key technology. Proceedings of the 2nd ACM Workshop on Security of Ad Hoc and Sensor Networks.

[3] Wong K., Zheng Y., Cao J., Wang S. A dynamic user authentication scheme for wireless sensor networks. Proceedings of the IEEE International Conference on Sensor Networks, Ubiquitous, and Trustworthy Computing.

[4] Tseng H., Jan R., Yang W. An improved dynamic user authentication scheme for wireless sensor networks. Proceedings of the Global Telecommunications Conference.

[5] Das M. (2009). Two-factor user authentication in wireless sensor networks. IEEE Trans. Wirel. Commun..

[6] He D., Gao Y., Chan S., Chen C., Bu J. (2010). An enhanced two-factor user authentication scheme in wireless sensor networks. Ad Hoc Sens. Wirel. Netw..

[7] Khan H., Alghathbar K. (2010). Cryptanalysis and security improvements of ‘two-factor user authentication in wireless sensor networks’. Sensors.

[8] Chen T., Shih W. (2010). A robust mutual authentication protocol for wireless sensor networks. ETRI J..

[9] Yuan J., Jiang C., Jiang Z. (2010). A biometric-based user authentication for wireless sensor networks. Wuhan Univ. J. Nat. Sci..

[10] Yoon E., Yoo K. A new biometric-based user authentication scheme without using password for wireless sensor networks. Proceedings of the 20th IEEE International Workshops on Enabling Technologies: Infrastructure for Collaborative.

[11] He D., Zhang Y., Chen J. (2012). Robust biometric-based user authentication scheme for wireless sensor networks. IACR Cryptol. ePrint Arch..

[12] Yoon E., Kim C. (2013). Advanced biometric-based user authentication scheme for wireless sensor networks. Sens. Lett..

[13] Choi Y., Lee Y., Won D. (2016). Security improvement on biometric based authentication scheme for wireless sensor networks using fuzzy extraction. Int. J. Distrib. Sens. Netw..

[14] Park Y., Lee S., Kim C., Park Y. (2016). Secure biometric-based authentication scheme with smart card revocation/reissue for wireless sensor networks. Int. J. Distrib. Sens. Netw..

[15] Koblitz N. (1987). Elliptic curve cryptosystems. Math. Comput..

[16] Miller V. (1985). Use of elliptic curves in cryptography. Adv. Cryptol..

[17] Dolev D., Yao A. (1983). On the security of public key protocols. IEEE Trans. Inf. Theory.

[18] Moon J., Choi Y., Jung J., Won D. (2015). An improvement of robust biometrics-based authentication and key agreement scheme for multi-server environments using smart cards. PLoS ONE.

[19] Choi Y., Lee D., Kim J., Jung J., Nam J., Won D. (2014). Security enhanced user authentication protocol for wireless sensor networks using elliptic curves cryptography. Sensors.

[20] Kocher P., Jaffe J., Jun B., Rohatgi P. (2011). Introduction to differential power analysis. J. Cryptogr. Eng..

[21] Das A. (2015). A secure and effective biometric-based user authentication scheme for wireless sensor networks using smart card and fuzzy extractor. Int. J. Commun. Syst..

[22] Wang C., Zhang X., Zheng Z. (2016). Cryptanalysis and improvement of a biometric-based multi-server authentication and key agreement scheme. PLoS ONE.

[23] Dodis Y., Kanukurthi B., Katz J., Smith A. (2013). Robust fuzzy extractors and authenticated key agreement from close secrets. IEEE Trans. Inf. Theory.

[24] Dodis Y., Reyzin L., Smith A. Fuzzy extractors: How to generate strong keys from biometrics and other noisy data. Proceedings of the International Conference on the Theory and Applications of Cryptographic Techniques.

[25] Das A. (2013). A secure and effective user authentication and privacy preserving protocol with smart cards for wireless communication. Netw. Sci..

[26] Von Oheimb D. The high-level protocol specification language hlpsl developed in the eu project avispa. Proceedings of the Applied Semantics 2005 Workshop.

[27] Avispa Tool Documentation Automated Validation of Internet Security Protocols and Applications. http://www.avispa-project.org/.

[28] Zhu H., Hao X. (2015). A provable authenticated key agreement protocol with privacy protection using smart card based on chaotic maps. Nonlinear Dyn..

[29] Nam J., Kim M., Park J., Lee Y., Won D. (2014). A provably-secure ECC-based authentication scheme for wireless sensor networks. Sensors.

[30] Park Y., Park Y. (2016). Three-factor user authentication and key agreement using elliptic curve cryptosystem in wireless sensor networks. Sensors.

